# Portable in situ temperature-dependent spectroscopy on a low-cost microfluidic platform integrated with a battery-powered thermofoil heater

**DOI:** 10.1002/viw.20220053

**Published:** 2023-01-11

**Authors:** Sai Krishna Katla, Wan Zhou, Hamed Tavakoli, Elvia Lilia Padilla Méndez, Xiujun Li

**Affiliations:** 1 Department of Chemistry and Biochemistry, University of Texas at El Paso, El Paso, Texas, USA; 2 Border Biomedical Research Center, & Forensic Science, University of Texas at El Paso, El Paso, Texas, USA; 3 Environmental Science and Engineering, University of Texas at El Paso, El Paso, Texas, USA

**Keywords:** curcumin, in situ spectroscopy, microfluidic devices, point-of-care analysis, temperature-dependent spectroscopy

## Abstract

A low-cost microfluidic platform integrated with a flexible heater was developed for in situ temperature-dependent spectroscopic measurement at the point of care. After verifying the system by comparing on-chip spectroscopic measurement of methylene blue with the conventional spectroscopy, we demonstrated its applications in temperature-dependent absorption spectroscopy of a model biomolecule, curcumin. The system is portable, battery-powered and requires ultra-low volumes of analytes, which is highly suitable for point-of-care characterization.

Advanced spectroscopic characterization techniques are constantly being explored to advance analytical measurement science and material characterizations.^1–3^ One of the challenges in spectroscopic characterization is to collect accurate and reliable data from samples in situ, thus providing timely feedback on certain reactions or dynamics of the system of interest. For instance, for carrying out temperature-dependent absorption spectroscopy, important data need to be collected within a fraction of seconds while heating the substance. Any mistake while doing so would require starting over the entire process again, making it a cumbersome and expensive ordeal. Further, the number of repeated trials could also be limited due to high costs and limited volumes of substances to be tested. Along with absorption spectroscopy, temperature-dependent spectroscopy has broad applications by using different types of spectroscopic detectors.^4–7^ For instance, Pietropaolo et al. reported the temperature dependence of the UV absorption spectra of biphenyl in solutions.^4^ Temperature-dependent Raman spectroscopy was used as a feasible tool to understand the thermal properties of two-dimensional materials (e.g., transition metal dichalcogenide) in photoelectronic devices.^5,6^ Most recently, multimodal variable-temperature scattering and spectroscopy have been applied to characterize and quantify temperature-dependent solution assembly of conjugated polymers, providing high-performance optoelectronics applications.^7^ Currently, although there are commercially available in situ spectroscopic techniques such as UV–visible, FT-IR, and X-ray absorption techniques that provide accurate spectroscopic information, expensive equipment, large volumes, and specially trained expertise are needed. Developing new techniques that are simpler and inexpensive could be the way to go for the analysis of samples in resource-limited settings.^8,9^

Microfluidic technologies have found a variety of applications in environmental monitoring,^10^ bioanalytical,^11^ and biomedical detection fields,^12^ such as nucleic acid analysis,^13–16^ protein detection,^17–19^ cellular analysis,^20^ cell culture,^21^ point-of-care (POC) diagnoses,^22–24^ and drug delivery.^25–27^ Microfluidic devices provide a promising platform with numerous features, including low-reagent consumption, miniaturization, portability, and low cost.^9^ Our lab previously developed a cost-effective battery-powered spectrophotometric system for POC analysis of nucleic acids using a microfluidic device,^2^ whereas it was not capable of in situ temperature-dependent spectroscopic measurement, mainly due to the lack of temperature control. Although different studies focused on using external heating sources for temperature control within microfluidic chips,^9,28,29^ they still faced challenges to achieve accurate measurements due to the temperature discrepancies between channels inside microfluidic devices and external heating sources and their related time lag. To the best of our knowledge, a low-cost and integrated microfluidic system for portable in situ temperature-dependent spectroscopic measurement in low-resource settings has not been reported yet.

Therefore, to address the issues above, we developed a novel low-cost microfluidic system integrated with a removable heating film that can be used to collect accurate in situ absorption spectroscopy data and carry out temperature-dependent spectroscopic analysis at the point of care. The microfluidic chip within this system requires an ultra-low sample volume of 10.6 μl for the analysis, making it advantageous in testing precious and limited amounts of samples. A flexible heating film has been integrated on the microfluidic device making our technique simple and cost-effective compared to those reported in previous literature,^30,31^ whereas the battery-powered feature further enhances its portability and capability for low-resources settings such as field detection.

The whole system contains a PMMA microfluidic chip, a thermofoil heater, battery power, and a portable spectrophotometer (USB-650-VIS-NIR, Ocean Optics) connected to a laptop via a USB cable, as shown in Figure (1A). The thermofoil heater is a thin and flexible heating film consisting of an etched-foil resistive heating element laminated between layers of polyimide. The thermofoil heater used in our experiments has a resistance of 5.5 Ω and was purchased from Minco (Minneapolis, MN, US). The heating film can be removed to be used in a different device.

The portable spectrophotometer used to characterize substances on the go does not need external AC power. It has a LED-boosted tungsten light source for the absorption wavelength range of 370–980 nm and a sample holder for 1 cm cuvettes. The microfluidic chip integrated with a thermofoil heater is tiny and can be placed within the cuvette holder, whereas the heater is connected to a battery power supply. The microfluidic chip was designed using Adobe Illustrator software and fabricated with PMMA by laser ablation using a laser cutter from Epilog Laser (Golden, CO, US), as we reported previously,^32^ and the detailed parameters are shown in Figure (1B). The microfluidic chip consisted of three PMMA layers (0.15 cm of thickness per layer) with a total dimension of 4.5 cm (*H*) × 1.32 cm (*W*) × 0.45 cm (*L*). The top layer contained a sample inlet, a sample outlet and sample introduction channels, and the middle layer had a circular sample well with a 0.3 cm diameter and 0.15 cm as depth (i.e., 10.6 μl of the total capacity). The bottom layer acted as a supporting base for the insertion of the thermofoil heater film. All PMMA layers were heat-bonded in an oven at 120°C for 60 min. Figure (1C) shows the photograph of a bonded PMMA microfluidic chip which includes all the three layers. Essentially, the inlet, the outlet, the sample well, and channels formed a V-shaped structure for sample introduction, in situ spectroscopic measurement, and waste collection. The sample well was the spectroscopic measurement area, and its location and size were optimized to align with the light path across the cuvette of the portable spectrophotometer. More experimental details are listed in the Supporting Information.

To integrate a flexible thermofoil heating film on the chip, the bottom surface of the middle PMMA layer and the top surface of the bottom PMMA layer were laser ablated to create a gap (~80 μm depth) that just holds the heating film (Figure 1B side-view). Figure (2A, B) shows the chip alongside the polyimide-based flexible thermofoil heater with a dimension of 4 cm (*L*) × 1.2 cm (*W*) × 0.05 cm (*H*) before and after the assembly, respectively. The chip was designed in such a way that it can accommodate the flexible battery-powered heater between the second and third layers (Figure 2B) and provide heat to the analyte in the sample well. Figure (2C) shows the schematic of the flexible heater assembled chip with the heater foil inserted in between the middle and the bottom layer of the chip. The flexible heater has a resistance of 5.5 Ω and can generate temperatures up to 100°C within the sample well. The heating function of the on-chip heater was then characterized by using a thermocouple. The plot between the temperature and the voltage in Figure (2D) shows a linear regression relationship between them, which indicates that different temperatures can be achieved by different voltages applied to the thermofoil heater and it could increase the sample temperature to 63°C with a voltage of 2.0 V.

After the characterization of the flexible heater integrated on the chip, the portable microfluidic spectroscopy system was tested by collecting visible light absorption spectrum of a common water-soluble dye, methylene blue (MB), and compared with a conventional spectroscopy method using a microplate reader. Figure (3A) shows the absorption spectra of a 10.6 μl MB sample with a concentration of 64.0 μM obtained by using the microfluidic spectroscopy system and a microplate reader. Both methods show similar spectra, with a well-defined characteristic peak of MB at 660 nm, indicating the feasibility of our microfluidic method for in situ spectroscopy. Furthermore, a series of standard solutions were measured using the microfluidic spectroscopy system to obtain the calibration curve. Figure (3B) shows the calibration curve obtained using different concentrations of MB ranging from 0 to 100 μM. The calibration curve is linear in the range from 0.5 to 100.0 μM, with an *R*^2^ value of 0.993. The LOD was calculated to be 0.93 μM, which was comparable to those of previously reported methods (i.e., 0.59^2^ and 1.1 μM^33^).

To study the in situ temperature-dependent absorption spectroscopy, curcumin was used as a model molecule as the absorption of curcumin is dependent on temperature, with many promising therapeutic applications owing to its antioxidant, anti-inflammatory properties, and antimicrobial effects.^34^ However, its low aqueous solubility and the consequent poor absorption hinder the effective and wide application of curcumin as biomedicine. Figure (4A) shows absorption spectra of curcumin at different temperatures (i.e., 25, 35, 45, 55, and 65°C) recorded using the in situ microfluidic spectroscopy. It can be seen that at lower temperatures, such as 25°C, curcumin exhibited poor absorbance. As the temperature increased, the absorbance showed a remarkable increase, and a well-defined peak around 425 nm was observed at 65°C. This trend of temperature-dependent absorbance changes is consistent with earlier reports.^34^ This can be understood from the fact that solubility of curcumin in water increases with an increase in temperature.^34^ In a neutral aqueous solution, curcumin is hydrophobic due to the lack of external polar functional groups as well as due to the conjugated backbone (see Figure 4B inset).^35^ It is likely that higher temperatures break intramolecular hydrogen bonding, leading to exposure of the polar groups to water and resulting in higher solubility at higher temperatures. Figure (4B) shows the relationship between the absorption intensity at 413 nm and different temperatures, which exhibits a linear relationship between the absorbance and the temperature from 25 to 65°C, with an *R*^2^ value of 0.989.

## CONCLUSION

In conclusion, a low-cost microfluidic spectroscopy system was successfully developed for portable in situ, low-volume, and temperature-dependent spectroscopy, and its spectroscopic applications have been demonstrated using MB at room temperature and curcumin at different temperatures. Compared to the conventional spectrophotometric system, the proposed system owns various merits, including portability, simplicity, low-cost, integrated battery-powered heating elements instead of external AC power, and low-reagent consumption (only 10.6 μl of analyte volume needed for the spectroscopic analysis). All these features allow the system ideal for wide applications such as precious chemical reaction characterization, kinetic studies of chemical reactions, and on-site spectroscopic measurement, especially when handling expensive chemicals as well as in low-resource settings.^36^

## Supplementary Material

SI

## Figures and Tables

**FIGURE 1:**
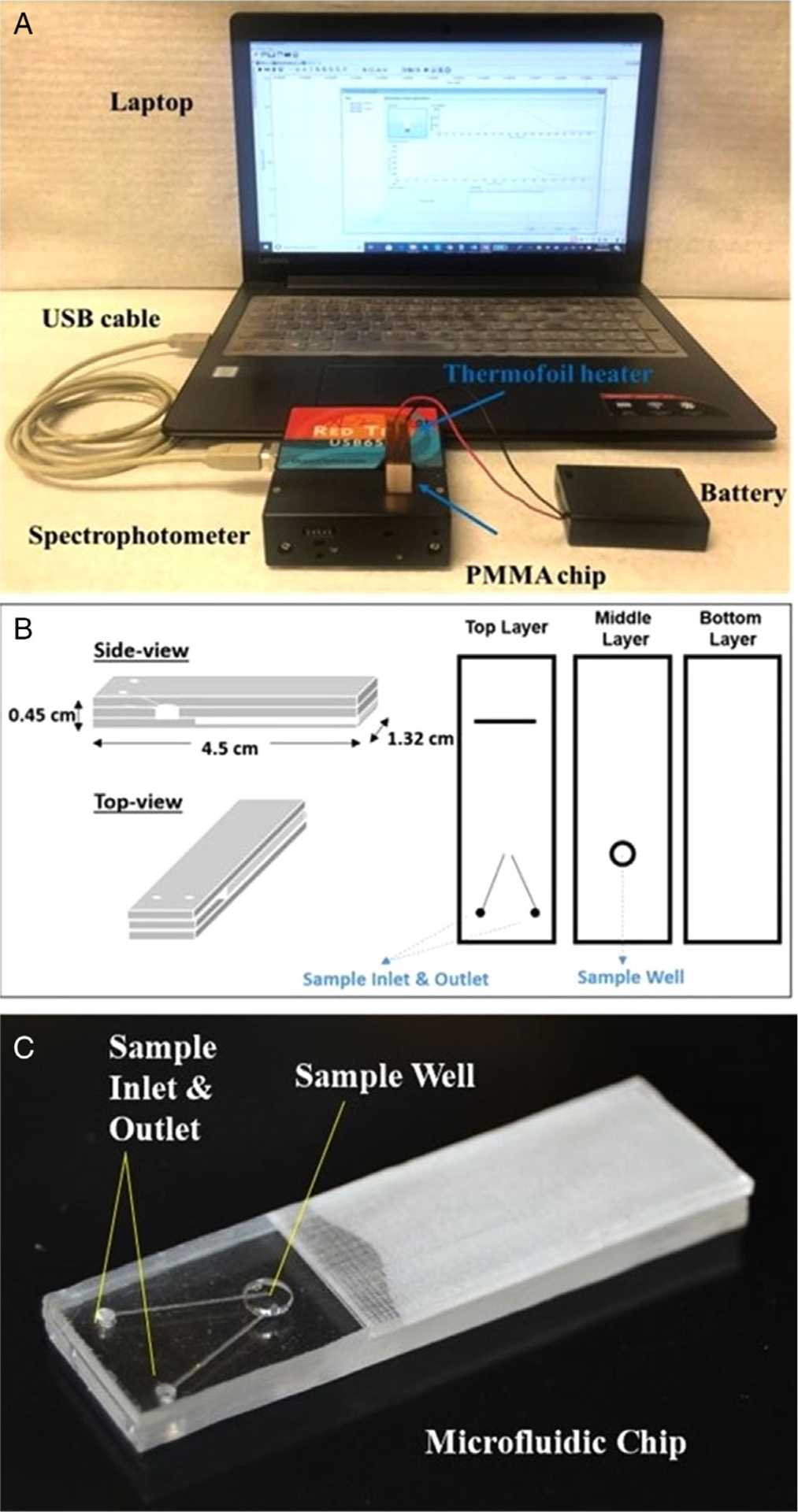
(A) Photograph of the microfluidic platform with a battery-powered thermofoil heater for in situ temperature-dependent spectroscopy. The heater is connected to the battery, and the spectrophotometer is connected to the computer via a USB cable. (B) Chip layout of an assembly (side-view and top-view) and the exploded view of the three different layers. The width and depth of the sample well were 0.3 and 0.15 cm, respectively. (C) Photograph of the microfluidic chip showing the sample well, sample inlet, and outlet

**FIGURE 2:**
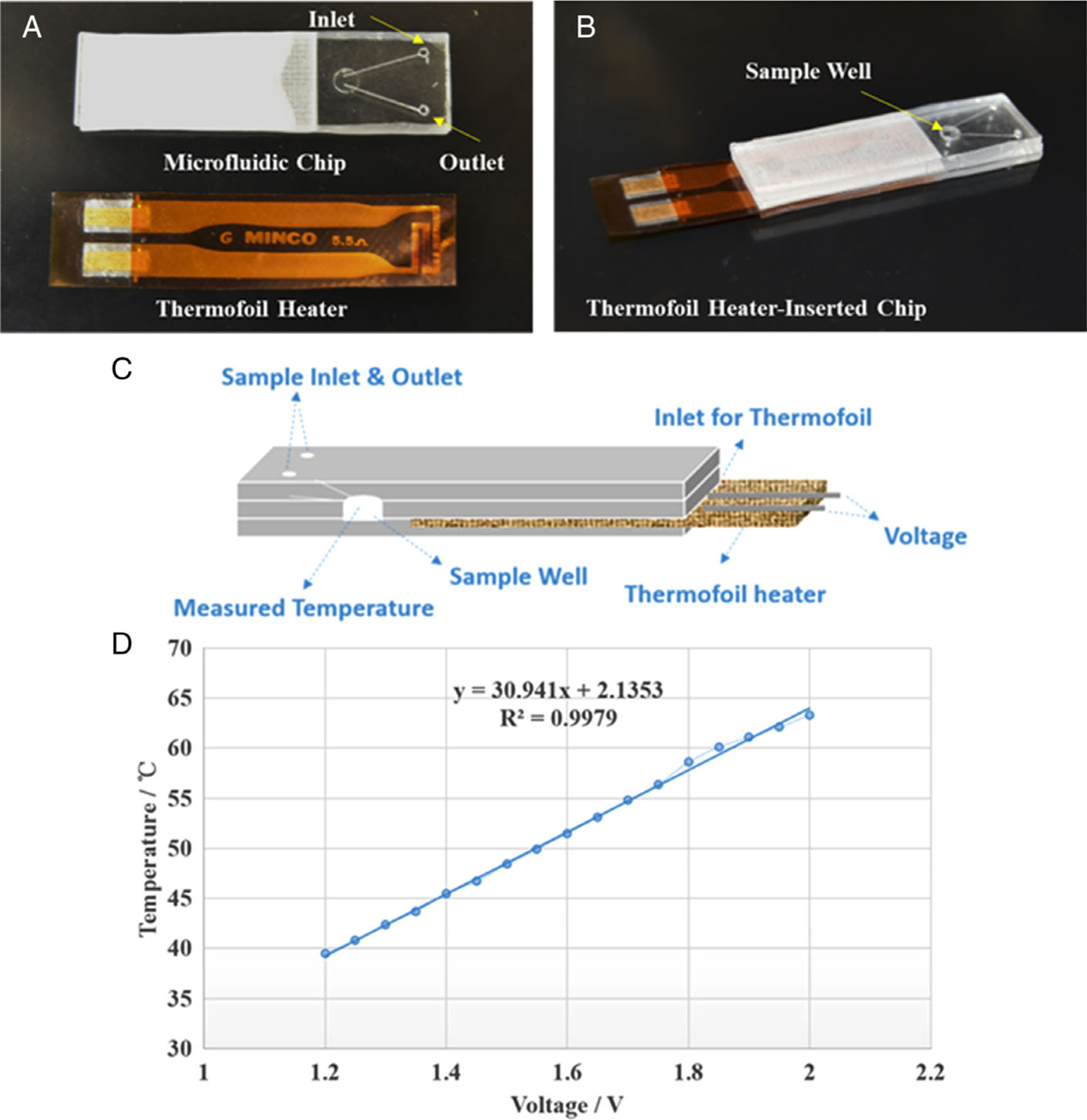
Integration of the thermofoil heater on the microfluidic chip: (A) the chip and the polyimide-based flexible heater; (B) flexible-heater assembled within the microfluidic chip; (C) schematic of cross-sectional view showing the thermofoil heater assembled chip; (D) temperature versus voltage curve obtained for the thermofoil heater (the distance between the thermofoil heater and the sample well is 0.2 cm)

**FIGURE 3:**
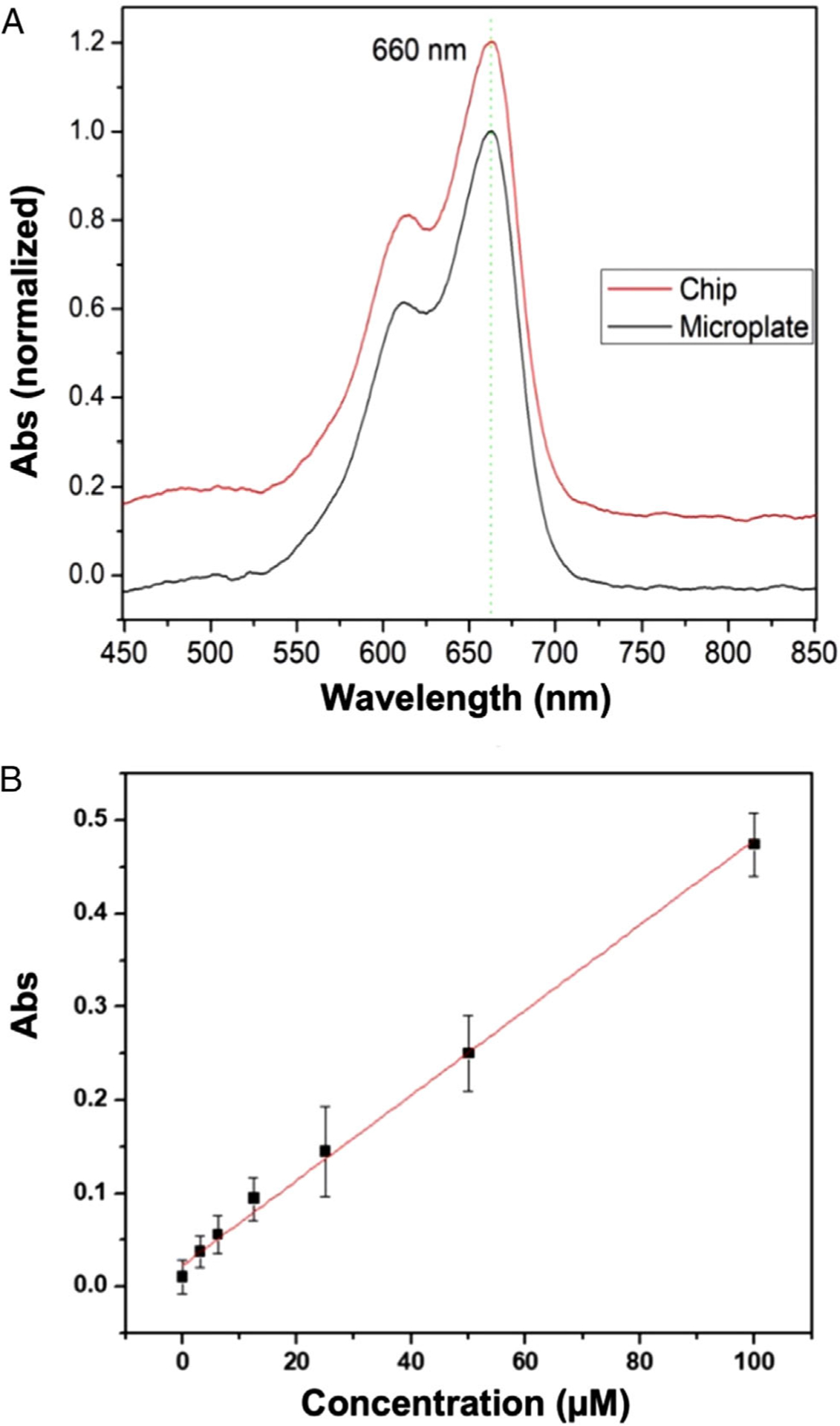
Characterization of the on-chip in situ spectroscopy using methylene blue (MB). (A) bUV–Vis spectrum comparison of 64 μM MB between a microplate reader (black) and the microfluidic spectroscopy system (red) at room temperature. Absorbance values from two different methods were normalized for easy comparison. (B) Calibration curve for the measurement of MB using the microfluidic spectroscopy system, showing linearity between absorbance at 660 nm versus MB concentrations (*y* = 0.00457*x* + 0.02309 with *R*^2^ = 0.993)

**FIGURE 4:**
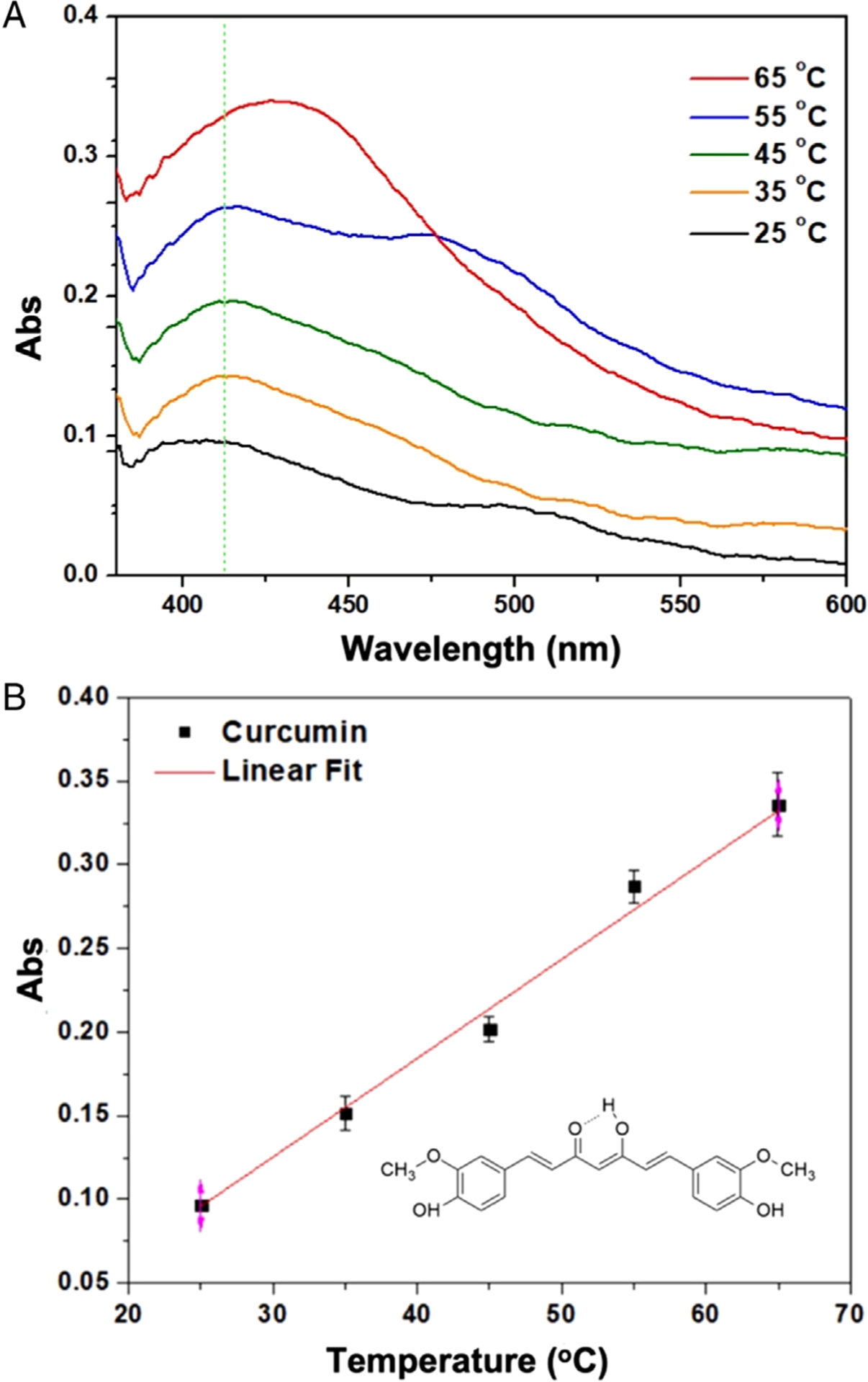
In situ temperature-dependent spectroscopy of curcumin using the portable microfluidic spectroscopy system: (A) visible-light absorption spectra of curcumin (100.0 μM) in water at different temperatures; (B) relationship between absorbance of curcumin at 413 nm and different temperatures ranging from 25 to 65°C (*R*^2^ = 0.989)
