# Supplementary material for: Portable in situ temperature-dependent spectroscopy on a low-cost microfluidic platform integrated with a battery-powered thermofoil heater
Source: View (Beijing). Author manuscript; Available in PMC 2024 Apr 1. (PMC10621267; doi:10.1002/viw.20220053)
Supplement: SI [file NIHMS1909489-supplement-SI.pdf]

Supplementary Information

Portable *in-situ* temperature-dependent spectroscopy on a low-cost microfluidic platform integrated with a battery-powered thermofoil heater

Sai Krishna Katla,[a,†] Wan Zhou,[a,†] Hamed Tavakoli,[a] Elvia Lilia Padilla Méndez[a] and XiuJun Li[a,b,c*]

[a]Department of Chemistry and Biochemistry, University of Texas at El Paso, 500 West University Ave, El Paso, Texas 79968, USA.

[b]Border Biomedical Research Center, & Forensic Science, University of Texas at El Paso, 500 West University Ave, El Paso, Texas 79968, USA.

[c]Environmental Science and Engineering, University of Texas at El Paso, 500 West University Ave, El Paso, Texas 79968, USA

† These authors contributed equally

Corresponding Author: XiuJun Li; Email: xli4@utep.edu

Experimental Section

Materials:

Poly (methyl methacrylate) (PMMA, 1.5 mm in thickness) Sheet was purchased from McMaster-Carr (Los Angeles, CA, US). Methylene Blue (MB), hydrochloric acid (37%), and sodium hydroxide (>97%) were purchased from Sigma-Aldrich (St. Louis, MO, US). Curcumin (>98%) was purchased from Alfa Aesar (Haverhill, MA, US). Water from Millipore Milli-Q system (18.2 MΩ·cm) was used in all our experiments. Unless otherwise stated, all other chemicals were of analytical grade and used as received.

Spectrophotometer USB650-VIS-NIR: The absorption spectroscopy of samples was recorded using the USB-650-VIS-NIR spectrophotometer device (Ocean Optics, Dunedin, Florida) and the data was collected using OceanView software. The spectrophotometer used is portable and can be used to characterize materials on the go. The system has absorption wavelength range of 370-980 nm. The spectrophotometer can be plugged-into a computer using a USB connector and is ready to use with the software. The spectrometer system includes a light source with a LED-boosted tungsten source and sample holder for 1 cm cuvettes.

Fabrication of PMMA-based microfluidic chip: A PMMA sheet (12"X12") was used in fabricating the microfluidic chip using a laser cutter (Zing, Epilog Laser). The design for the chip was made using Adobe Illustrator software and the channels in the chip were cut using the laser cutter. The dimensions of the chip are 4.5 cm x 1.32 cm x 0.45 cm. The chip consists of three layers; each layer with the same dimensions 4.5 cm x 1.32 cm x 0.15 cm, with a top layer

containing the channels to introduce the reagents, a middle layer containing the sample well for spectroscopic measurement, and a bottom layer to seal the well.

Methylene blue absorption spectroscopy for calibration curve

A stock solution of 100 μM methylene blue (MB) was initially prepared using 3.2 mg in 100 mL of Milli-Q water. The stock solution was used in the preparation of 8 different dilutions from 0 to 100 μM. To analyze the absorption calibration curve of the MB, 10.6 μL of each of the individual dilutions were separately added to a sample well in the PMMA chip. A USB-650-VIS-NIR portable spectrophotometer (Ocean Optics, Dunedin, Florida, US) was used in recording the spectra of methylene blue samples in the PMMA chip. The spectrophotometer used OceanView software to acquire the spectra. In comparison, MB solutions were also characterized via a microplate reader (Molecular Devices, Sunnyvale, CA, US) to obtain UV-vis spectra.

In-situ temperature-dependent absorption spectroscopy of curcumin

Curcumin (100 μM) was prepared using Milli-Q water and 10.6 μL of the sample was transferred to the sample well on the microfluidic chip. The chip has a flexible heater (4 cm X 1.2 cm X 0.05 cm) inserted between the second and third layer of the chip. This flexible heater was supplied with voltage from battery power to increase the temperature of curcumin in the sample well of the chip. Curcumin was heated to different temperatures (25 ºC, 35 ºC, 45 ºC, 55 ºC and 65 °C) and the absorption spectra were simultaneously collected using a portable USB-650-VIS-NIR spectrophotometer.
